# Effect of a participatory organizational workplace intervention on workplace social capital: post-hoc results from a cluster randomized controlled trial

**DOI:** 10.1186/s12889-019-6903-1

**Published:** 2019-06-06

**Authors:** Elisabeth Framke, Ole Henning Sørensen, Jacob Pedersen, Thomas Clausen, Vilhelm Borg, Reiner Rugulies

**Affiliations:** 1 National Research Centre for the Working Environment, Lersø Parkallé 105, DK-2100 Copenhagen, Denmark; 2 Department of Public Health, University of Copenhagen, Øster Farimagsgade 5, DK-1014 Copenhagen, Denmark; 3 Department of Psychology, University of Copenhagen, Øster Farimagsgade 2A, DK-1353 Copenhagen, Denmark

**Keywords:** Psychosocial, Workplace social capital, Vertical, Horizontal, Occupational health, RCT

## Abstract

**Background:**

A high level of workplace social capital (WSC) may contribute to the protection of employees’ health. We hypothesized that a participatory workplace intervention would increase the level of WSC defined as vertical WSC (i.e. WSC linking together employees and their leaders) and horizontal WSC (i.e. WSC bonding employees together).

**Methods:**

We conducted a secondary data analysis of a cluster randomized controlled trial that was implemented among all employees in 78 municipal Danish pre-schools (44 intervention and 34 control group schools). The study sample consisted of 606 employees, 386 in the intervention and 220 in the control group. The intervention aimed to improve the psychosocial working environment by using a participatory approach and focusing on core job tasks. Vertical and horizontal WSC was measured by five and four items, respectively, at baseline and at 24-months follow-up. We estimated intervention effect by calculating the interaction of change over time by group assignment (intervention versus control group) and included workplace identification number in a repeated statement to take into account that employees were nested within workplaces. We conducted post-hoc analyses to examine whether intervention effect differed by implementation degree.

**Results:**

WSC decreased in both groups. In the main analyses, there was no statistically significant difference between intervention and control group, neither for vertical nor horizontal WSC. However, when we excluded intervention workplaces with a low degree of implementation, we found a statistically significant difference between the intervention and the control group (estimate: 0.25, 95% CI: 0.00 to 0.50, *p* = 0.049), indicating that vertical WSC decreased in the control group and remained stable in the intervention group.

**Conclusions:**

There was not a statistically significant difference between intervention and control group in the main analysis. Post-hoc analyses, however, suggest that the intervention may have prevented a decrease in vertical WSC among employees in workplaces with a high or a medium degree of implementation.

A conference abstract with the key results of this study has been previously presented and published, European Journal of Public Health, Volume 28, Issue suppl_4, November 2018, cky260, https://academic.oup.com/eurpub/article/28/suppl_4/cky260/5187184.

**Trial registration:**

ISRCTN16271504, retrospectively registered on November 15, 2016.

## Background

Employees’ involvement, knowledge, and ownership have been shown to be important factors for the success of organizational workplace interventions to improve employees’ health [1, 2]. The participatory approach implies that employees take an active part in the workplace problem analysis and solution finding process. The participatory approach has the potential to increase employees’ involvement, commitment, and job control and has further the potential to create intervention activities that are tailored to the specific needs of the workplace. This dual benefit of participation was emphasized by Aust and Ducki [3] as an important intervention component of the health circle approach.

A high level of workplace social capital (WSC) may contribute to the protection of employees’ health and wellbeing [4–6]. Social capital refers to beneficial resources in relations between people [7]. WSC refers to beneficial resources in relations between people at work, i.e. in relations between employees and in relations between employees and leaders [8, 9].

Assuming that the research findings on the beneficial effects of WSC on employees’ health and wellbeing are valid, identifying and implementing workplace interventions that increase WSC levels would be important for protecting and promoting employees’ health and wellbeing. However, little is known if workplace interventions can affect WSC levels.

The aim of this study was therefore to examine whether a participatory organizational workplace intervention had an effect on the level of WSC. To this end, we performed a secondary data analysis of a cluster-randomized controlled trial in Danish, municipal pre-schools. The original aim of this trial was to study whether the intervention had an impact on employees’ well-being and sickness absence. Results on these primary endpoints are published elsewhere [10, 11]. Change in WSC was not a defined endpoint of the trial. However, because the trial was based on the participatory approach and included several activities that may have affected WSC by increasing resources that enhanced better relations at work we deemed it reasonable to assume that the intervention may have led to an increase in WSC. Activities that might have increased relational resources at work and thereby affected WSC were building steering groups consisting of a leader and two employee representatives that were responsible for developing and implementing workplace specific intervention activities while involving all employees, workplace culture and change management training. Examples of workplace specific intervention activities were improving communication and professional feedback; changes in allocation of overtime, work schedules and holiday schedules; re-organization of staff meetings to advance professional reflection; modifications to work culture; and re-organization of physical indoor and outdoor environment [12].

Based on the assumption that this participatory workplace intervention may had led to an increased level of WSC, we test the hypothesis, that employees in intervention group workplaces compared to employees in control group workplaces would report a greater increase in WSC defined as vertical WSC (i.e. WSC linking together employees and their leaders) and horizontal WSC (i.e. WSC bonding employees together). In addition to testing this hypothesis, we also conducted post-hoc analyses to examine whether the intervention effect differed by implementation degree.

## Methods

This study is based on data from an intervention study called the Pioneer intervention study. The Pioneer intervention was conducted by work environment consultants from a private company among all employees in 78 municipal pre-schools in the Children and Youth Administration in the Municipality of Copenhagen [10–12]. The aim of this intervention was to study the effect of a participatory workplace intervention in municipal pre-schools. Employees in pre-schools have compared to other groups of employees in Denmark a high level of sickness absence [10]. The intervention was initiated by the Municipality of Copenhagen in Denmark. About 90% of all 1–2 years old and about 97% of all 3–5 years old children attend pre-schools. About 70% of Danish pre-schools are run by municipalities. The remaining pre-schools are run by private organizations [11, 12].

### Study design and participants

**Fig. 1:**
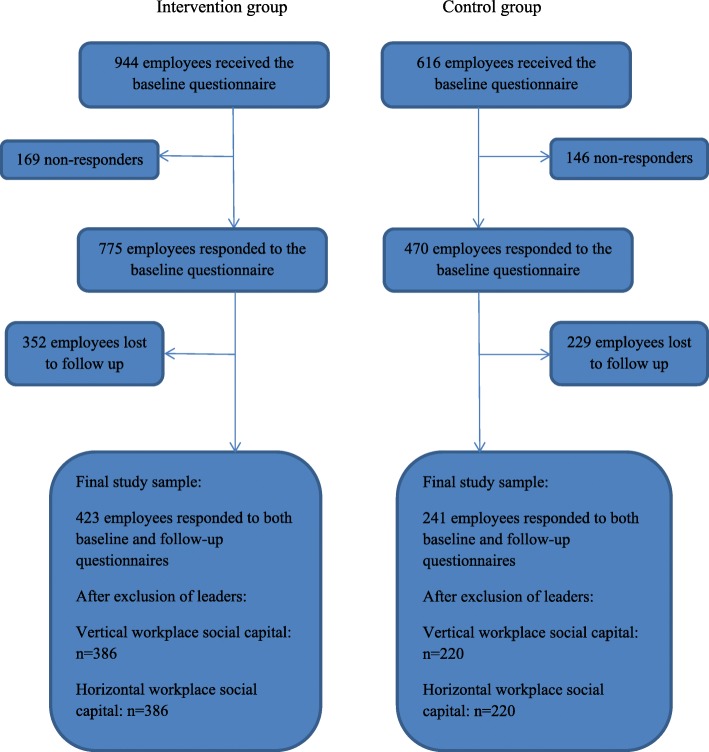
Flow chart towards the final study sample

The intervention targeted the organizational level therefore the randomization was performed as a cluster randomization at the workplace level. Resources were available to implement the intervention at 44 of the 78 workplaces, with the remaining 34 workplaces serving as the control group. A statistician performed the randomization accordingly. Of the 78 workplaces, seven were lost to follow-up, three in the intervention and four in the control group. Therefore, analyses in this article are based on 41 intervention group workplaces and 30 control group workplaces. Figure 1 shows the flow chart towards the final study sample, including participants lost to follow-up. We excluded pedagogical leaders, because their WSC may differ from the WSC of the employees, yielding a final study of 606 participants, 386 in the intervention, and 220 in the control group. These participants worked as nursery nurses (*n* = 354), nursery nurse assistants (*n* = 194) or in other job groups (*n* = 58, e.g., kitchen staff and school caretakers).

According to Danish law, research studies that use solely questionnaire and register data do not need approval from the National Committee on Health Research Ethics (Den Nationale Videnskabetiske Komité).

### The intervention

The purpose of the intervention was to improve the psychosocial work environment by focusing on core job tasks, which consequently should improve employee well-being and reduce risk of short-term sickness absence. The intervention was targeted the organizational level, i.e. targeted at changing aspects of work rather than individuals. Examples of this type of interventions are job redesign, implementation of autonomous teams, rearranging working- and resting times, improving communication, and increasing social support [13, 14].

In addition to the organizational approach, the participatory approach and the core job task focus were key elements of the intervention. The intervention content is described in detail elsewhere [15]. Briefly, the cluster randomization was performed in June 2011. The intervention was introduced to steering group members, i.e. the leader and two employee representatives, and employees in September 2011. Intervention activities were finalized in June 2013.

Participants’ involvement in the development and implementation of activities tailored to the local needs of the workplaces was pivotal in this intervention. Steering group members participated in seminars and workshops on how to develop and implement intervention activities while involving employees, change management training, workplace culture, and a how to evaluate workplace changes. Steering group members and employees received support from work environment consultants during the complete intervention period. Based on seminars and consultants’ support, steering group members and employees developed and implemented workplace specific activities with a focus on improving performance of core job tasks. The joint involvement of leader and employees in this type of participatory intervention is assumed to increase relational resources at workplaces.

### Effect measures

We measured WSC with self-administered questionnaires at baseline (September 2011) and at follow-up 24 months later. Of the nine WSC items, five were derived from the Danish Work Environment Cohort Study (DWECS) from the year 2010 [16], one was derived from DWECS from the year 2005 [17], one was derived and slightly modified from Gittell’s questionnaire on relational coordination [18], and two items were formulated for the purpose of this study. A factor analysis (rotation method: varimax) showed two distinct factors with eigenvalues of 4.14 and 1.78, respectively. We named these factors “vertical WSC” (i.e. social capital linking together employees and their leaders, five items) and “horizontal WSC” (i.e. social capital bonding employees together, four items) in accordance with theoretical considerations about different types of social capital in the literature [8]. All rotated factor loadings were > 0.70 for vertical WSC and > 0.65 for horizontal WSC. Cronbach’s alpha was 0.87 and 0.80 for vertical and horizontal WSC, respectively.

Appendix 1 shows the items and response categories for the two WSC scales. Participants were included if they responded to at least three of the five items of the vertical component of WSC and to at least two of the four items of the horizontal component of WSC. Response categories went from ‘To a very small extent’ [1] to ‘To a very large extent’ [5].

For each of the 71 workplaces we calculated the workplace aggregated mean score of the two measures of WSC at baseline and at follow-up. Then, we assigned the workplace aggregated mean scores to all individual participants within each of the 71 workplaces. Intra-class correlations were 0.35 and 0.14 for vertical and horizontal WSC, respectively.

### Degree of implementation measure

Appendix 2 shows the items and response categories for the degree of implementation measure. We measured the degree of implementation using three items from self-administered questionnaires at follow-up (Cronbach’s alpha was 0.86). Intervention group participants were asked to evaluate to what extent (i) they had influence on intervention activities; (ii) they participated in intervention activities; and (iii) their closest leader supported intervention activities [19]. Participants rated the three items on a five-point scale (5, To a very high degree, 4, To a high degree, 3, Partly, 2: To a low degree; 1: To a very low degree). Each of the three items was aggregated at the workplace level. We then calculated the mean score of the three workplace level measures, yielding a workplace level continuous measure of degree of implementation. Then, we dichotomized this continuous measure, resulting in a) one group of participants within 27 workplaces with a high or medium degree of implementation, and b) one group of participants within 14 workplaces with a low degree of implementation. See Appendix 2 for details on the dichotomization process.

### Statistical analysis

All analyses were conducted using SAS 9.4 statistical software.

We tested baseline differences between the intervention and the control group with regard to employee and workplace characteristics and baseline scores of the two measures of WSC using Chi-square test and two sample t-test.

We then calculated baseline and follow-up mean scores for each of the two WSC measures separately for the two groups. For each WSC measure, we analyzed changes from baseline to follow-up separately for the two groups using paired t-test.

Next, we estimated the intervention effect by calculating the interaction of change over time by group assignment (intervention group vs. control group) using the GENMOD procedure and included workplace identification number in a repeated statement to take into account that employees were nested within workplaces. We calculated unadjusted estimates, estimates adjusted for sex and age (continuous) (Model 1), and estimates with further adjustments for job group, workplace type and – size, and baseline scores of endpoints (Model 2).

We conducted additional analyses based on individual-level assessments instead of workplace aggregated mean scores where we repeated the analyses on within group changes and the analyses on interaction change x group.

Finally, we conducted post hoc analyses on within group changes and on interaction change times group by analyzing separately intervention group workplaces with a high/medium and with a low degree of implementation.

## Results

### Characteristics of participants

Table 1 shows the characteristics of the intervention and the control group in the study sample. Intervention group employees were statistically significantly younger (42.4 versus 44.6) and worked at workplaces greater in size (23.4 employees versus 21.8 employees). There was not a statistically significant difference between the two groups with regard to sex, job group, or workplace type. The two groups differed statistically significantly with regard to baseline scores of vertical and horizontal WSC with the intervention group showing higher scores (3.87 versus 3.71 for vertical and 4.00 versus 3.86 for horizontal WSC). Repeating the comparison of baseline scores of the two measures of WSC using individual assessments, instead of workplace aggregated mean scores yielded similar the results (data is shown in Table 4 in Appendix 3).

**Table 1:** Employees’ and workplaces’ characteristics and baseline scores of workplace social capital in the intervention and the control group in the study sample

	Intervention group				Control group				Chi^2^ (p)	t (p)
	Mean	SD	%	n	Mean	SD	%	n		
Employee characteristics				386				220		
Age	42.4	10.4			44.6	9.8				**2.53 (0.01)**
Women			86.0	332			89.5	197	1.58 (0.21)	
Job group									2.13 (0.34)	
Nursery nurses			60.4	233			55.0	121		
Nursery nurse assistants			31.1	120			33.6	74		
Other job groups			8.5	33			11.4	25		
Workplace characteristics										
Size	23.4	8.4			21.8	9.6				−**1.98 (0.05)**
Workplace type									1.82 (0.40)	
Integrated			76.9	297			78.6	173		
Day care			18.7	72			19.1	42		
Kindergarten			4.4	17			2.3	5		
Baseline scores of social capital										
Vertical	3.87	0.43		386	3.71	0.52		220		−**3.79 (< 0.01)**
Horizontal	4.00	0.32		386	3.86	0.22		220		−**6.58 (< 0.01)**

Statistically significant results are printed in bold

### Effect of the intervention on WSC

Table 2 shows changes in the two WSC scales, based on workplace-mean WSC scores, from baseline to follow-up separately for intervention and control group. Vertical WSC decreased in both groups (− 0.14 in the intervention group, − 0.16 in the control group, *p* < 0.01 in both groups). Horizontal WSC decreased in the intervention group (− 0.08, *p* < 0.01) but not in the control group (*p* = 0.69). Repeating the analyses with WSC scales based on individual-level assessments of WSC yielded similar results (data is shown in Table 5 in Appendix 4).

**Table 2:** Within group changes in workplace social capital during 24 months of follow-up

	Intervention group						Control group					
	n	Baseline Mean (SD)	Follow-up Mean (SD)	change	t	*p*	n	Baseline Mean (SD)	Follow-up Mean (SD)	change	t	*p*
Social capital												
Vertical	386	3.87 (0.43)	3.73 (0.44)	−0.14	6.20	**<.01**	220	3.71 (0.52)	3.56 (0.59)	−0.16	3.74	**<.01**
Horizontal	386	4.00 (0.32)	3.92 (0.32)	−0.08	6.26	**<.01**	220	3.86 (0.22)	3.85 (0.33)	−0.01	0.40	0.69

Statistically significant results are printed in bold

Table 3 shows the analyses on the intervention effect, i.e. the interaction of change in WSC, based on workplace-mean WSC scores, from baseline to follow-up times group assignment. There was no statistically significant difference between the intervention and the control group, neither for vertical nor horizontal WSC, and neither in the unadjusted nor in the adjusted analyses. Repeating the analyses using individual-level assessments of WSC yielded similar results (data is shown in Table 6 in Appendix 4).

**Table 3:** Intervention effect on workplace social capital in the intervention group compared to the control group during 24 months of follow-up

	n	Unadjusted			Model 1			Model 2		
		Est	95% CI	*p*	Est	95% CI	*p*	Est	95% CI	*p*
Social capital										
Vertical	606	0.02	−0.27-0.31	0.89	0.02	− 0.27-0.31	0.90	0.07	−0.18-0.32	0.58
Horizontal	606	−0.08	−0.21-0.05	0.22	−0.08	− 0.21-0.05	0.24	− 0.04	−0.16-0.09	0.58

Interaction change x group analyses: Unadjusted; Model 1: adjusted for sex and age (continuous); Model 2: further adjusted for job group (nursery nurse, nursery nurse assistant, other job group), workplace type (integrated, day care, kindergarten), workplace size (continuous), and baseline values of endpoints. Workplace identification number is included in a repeated statement

### Post hoc analyses

Results from post-hoc analyses that took the degree of the implementation into account are shown in Appendix 5. Within group analyses showed that vertical WSC decreased in the intervention workplaces with a low degree of implementation (− 0.36, *p* < 0.01) but not in the intervention workplaces with a high/medium degree of implementation. Horizontal WSC decreased both in the intervention workplaces with a high/medium and a low degree of implementation (Table 7 in Appendix 5).

When we compared control group workplaces with intervention group workplaces with a high/medium degree of implementation, we found a statistically significant more favourable change in vertical WSC in the intervention group (*p* = 0.049, Table 8 in Appendix 5). In contrast, when we compared control group workplaces with intervention group workplaces with a low degree of implementation, we found a statistically non-significant less favourable change in vertical WSC in the intervention group (*p* = 0.15, Table 9 in Appendix 5). There was no intervention effect on horizontal WSC (Table 8 in Appendix 5).

## Discussion

The hypothesis that this participatory workplace intervention would lead to that employees in intervention group workplaces compared to employees in control group workplaces would report a greater increase in WSC was not confirmed. There was no statistically significant difference between the two groups during the 24-months follow-up period. Post-hoc analyses showed, however, a statistically significant difference between the intervention group with a high/medium degree of implementation and the control group with regard to vertical WSC. Vertical WSC remained stable in the intervention group and deteriorated in the control group. Post-hoc analyses showed no intervention effect on horizontal WSC.

Within group changes from baseline to follow-up revealed a decrease in vertical WSC in both groups and in horizontal WSC in the intervention group only. When stratifying for implementation degree, we found a notable decrease in vertical WSC in the intervention group with a low degree of implementation as opposed to the intervention group with a high or medium degree of implementation. Based on the combined findings from main and post-hoc analyses, we suggest that implementing this participatory workplace intervention to a high or medium degree may have prevented a decrease in vertical WSC compared to the control group (change of − 0.02 versus − 0.16 points). In contrast, implementing this participatory workplace intervention to a low degree may have resulted into an even larger decrease in vertical WSC in the intervention group compared to the control group (change of − 0.36 versus − 0.16 points). Thus, a poor implementation of the intervention may have had an adverse effect on vertical WSC. We can only speculate about the reasons for such a possible adverse effect, but it is known from the literature that poorly implemented interventions may cause disappointment in employees and that such disappointment can result into a decrease of the quality of the psychosocial work environment [20]. The degree of implementation did not affect horizontal WSC.

We do not know what caused the decrease in vertical WSC from baseline to follow-up in. One possible reason could have been the introduction of changes in the general management structure in municipal pre-schools at the time just before this intervention study was initiated. Another possible reason could have been the Municipality of Copenhagen’s strong focus on sickness absence in this time period, including the implementation of mandatory sickness absence meetings for pre-school employees.

With regard to intervention group workplaces qualitative process evaluation showed both supportive mechanisms and hindrances associated with workplaces’ readiness for change and the organizational fit of the intervention depending on workplaces appraisal of the intervention [21]. Some participants with a negative appraisal of the intervention experienced the intervention as something unwanted, and they felt patronized by it. Further, some participants felt that there were no major problems at their workplace and that therefore the intervention was a waste of their time. Other participants with a positive appraisal of the intervention emphasized that an important advantage of the intervention was that it enabled them to adjust the workplace specific intervention activities to suit the needs of their workplace [21].

Previously, we found similar results in terms of illegitimate job tasks where this intervention did not improve the psychosocial workplace factors but protected against deterioration [15]. Protection against an increase in adversity instead of reduction in adversity has also been reported in other psychosocial intervention studies [22, 23]. In some cases it might be more realistic to aim for preventing deterioration rather than improving working conditions. This said, though, it should be noted that there were also important differences between our earlier study on illegitimate job tasks and the current study on WSC. Illegitimate tasks were, unlike WSC, a primary target of the intervention (that had a focus on core job tasks). Further the intervention effect on illegitimate job tasks was found in the main analysis and not in a subpopulation where employees with low degree of implementation workplaces were excluded [15].

We had assumed that a participatory workplace intervention could have a significant effect on WSC in a favorable direction due to building steering groups consisting of a leader and employee representatives that were responsible for developing and implementing local intervention activities while involving all employees. Additionally, steering group members received training within amongst others change management training, and workplace culture. In line with that, our measure of implementation degree was based on items regarding management support and employee participation. It is notable, that if implemented to a medium or high degree, the intervention appears to have protected against adversity regarding linking employees and their leaders together (vertical WSC) but not regarding bonding together employees (horizontal WSC). However, it should be noted that it is not clear how big a change in WSC is needed in order to affect employees’ health and wellbeing.

### Strengths and limitations

The main strength of this study is the RCT design including 78 workplaces, of which it was possible to conduct analyses within 71 workplaces. Further, the intervention was implemented by professional work environment consultants, of which one consultant managed the implementation and secured that all workplaces received the same overall intervention. Finally, a strength of the study was that vertical and horizontal WSC was measured with five and four items, respectively, with a Cronbach’s alpha of 0.87 (vertical WSC) and 0.80 (horizontal WSC).

A limitation of the study is the rather long follow-up period of 24 months. Ideally, when implementing comprehensive workplace interventions of a long duration, endpoints and implementation degree should be measured not just before and after the intervention but also during the intervention with at least one additional measurement between baseline and follow-up. Further, it is a limitation that implementation degree was assessed with a rather simple measure, consisting of just three self-reported items. Other intervention studies have used more comprehensive measures for the degree of implementation [24, 25].

## Conclusion

There was not a statistically significant effect of the intervention on WSC in the main analysis. Post-hoc analyses, however, suggest that the intervention may have prevented a decrease in vertical WSC among employees in workplaces with a high or a medium degree of implementation.

## Appendix 1

### Items for measuring vertical and horizontal workplace social capital

Items for measuring vertical workplace social capital:

- We have confidence in the management
- The management trusts us to do our work well
- Our immediate manager contributes to that we can achieve the best possible result
- Our immediate manager treats us with respect and dignity
- Are employees involved in decisions regarding workplace changes?

Participants were included, if they responded to at least three of the five items on vertical workplace social capital.

Items for measuring horizontal workplace social capital:

- We help each other in achieving the best possible result
- The cooperation between colleagues with different educational backgrounds is good
- Do different groups of employees respect each other’s work?
- Is the work distributed fairly?

Participants were included, if they responded to at least two of the four items on horizontal workplace social capital.

Response categories to all nine items:

5 = To a very large extent; 4 = To a large extent; 3 = Somewhat; 2 = To a small extent; 1 = To a very small extent.

For each of the workplaces we calculated a workplace mean score of the two measures of WSC at baseline and at follow-up. Then, we assigned the workplace mean scores to all individual participants within each of the workplaces. Intra-class correlations were 0.35 and 0.14 for vertical and horizontal WSC, respectively, meaning that 35 and 14% of the variance in the individual level vertical and horizontal WSC, respectively, could be explained by workplace.

## Appendix 2

### Items for measuring the degree of implementation

- Have you had influence on the activities in the Pioneer intervention?
- Have you participated in the activities in the Pioneer intervention?
- Has your closest leader supported the activities in the Pioneer intervention?

Response categories to all three items:

5 = To a very high degree; 4 = To a high degree; 3 = Partly; 2 = To a low degree; 1 = To a very low degree.

Dichotomization:

We calculated the mean score of the three items and then dichotomized the score into “high or medium degree of implementation” and “low degree of implementation”. If the mean workplace level score was ≥3 (i.e. ≥ “partly”) then the workplace was recorded with a high or medium high degree of implementation. If the mean workplace level score was < 3 (i.e. <“partly”), then the workplace was recorded with a low degree of implementation.

## Appendix 3

### Baseline scores of workplace social capital based on individual-level assessment of workplace social capital

**Table 4:** Baseline scores of workplace social capital in the intervention and the control group in the study sample based on participants’ own assessments

	Intervention group			Control group			t (p)
	Mean	SD	n	Mean	SD	n	
Baseline scores of social capital							
Vertical	3.87	0.68	378	3.70	0.79	212	**−2.63 (0.01)**
Horizontal	4.00	0.58	376	3.86	0.65	217	**−2.85 (<.01)**

Statistically significant results are printed in bold

## Appendix 4

### Within group changes in workplace social capital and intervention effect on workplace social capital based individual-level assessment of workplace social capital

**Table 5:** Within group changes in workplace social capital (WSC) during 24 months of follow-up based on individual-level assessment of WSC

	Intervention group						Control group					
	n	Baseline Mean (SD)	Follow-up Mean (SD)	change	t	*p*	n	Baseline Mean (SD)	Follow-up Mean (SD)	change	t	*p*
Social capital												
Vertical	378	3.87 (0.68)	3.73 (0.71)	−0.14	3.70	**< 0.01**	212	3.70 (0.78)	3.56 (0.86)	−0.14	2.00	**0.047**
Horizontal	376	4.00 (0.58)	3.92 (0.62)	−0.08	2.68	**0.01**	217	3.86 (0.65)	3.85 (0.65)	−0.01	0.14	0.89

Statistically significant results are printed in bold

**Table 6:** Intervention effect on workplace social capital (WSC) in the intervention group compared to the control group during 24 months of follow-up based on individual-level assessment of WSC

	n	Unadjusted			Model 1			Model 2		
		Est	95% CI	*p*	Est	95% CI	*p*	Est	95% CI	*p*
Social capital										
Vertical	590	−0.00	−0.28 – 0.28	0.99	0.00	−0.27 – 0.27	1.00	0.08	−0.15 – 0.36	0.48
Horizontal	593	−0.08	−0.21 – 0.05	0.24	−0.07	− 0.20 – 0.06	0.28	0.00	−0.13 – 0.13	0.99

Interaction change x group analyses: Unadjusted; Model 1: adjusted for sex and age (continuous); Model 2: further adjusted for job group (nursery nurse, nursery nurse assistant, other job group), workplace type (integrated, day care, kindergarten), workplace size (continuous), and baseline values of endpoints. Workplace identification number is included in a repeated statement

## Appendix 5

### Within group changes in workplace social capital and intervention effect on workplace social capital while taking degree of implementation into account

**Table 7:** Within group changes in workplace social capital during 24 months of follow-up, stratified by degree of implementation

	Intervention group: high/medium degree of implementation						Intervention group: low degree of implementation					
	n	Baseline Mean (SD)	Follow-up Mean (SD)	change	t	*p*	N	Baseline Mean (SD)	Follow-up Mean (SD)	change	t	*p*
Social capital												
Vertical	253	3.93 (0.45)	3.91 (0.33)	−0.02	0.77	0.44	133	3.76 (0.36)	3.40 (0.41)	−0.36	9.72	**<.01**
Horizontal	253	4.08 (0.29)	3.99 (0.29)	−0.09	5.07	**<.01**	133	3.86 (0.32)	3.78 (0.34)	−0.08	3.66	**<.01**

Statistically significant results are printed in bold

**Table 8:** Intervention effect on workplace social capital in the intervention group (high/medium degree of implementation) compared to the control group during 24 months of follow-up

	n	Unadjusted			Model 1			Model 2		
		Est	95% CI	*p*	Est	95% CI	*p*	Est	95% CI	*p*
Social capital										
Vertical	473	0.14	−0.15-0.44	0.33	0.15	−0.14-0.43	0.32	0.25	0.00–0.50	**0.049**
Horizontal	473	−0.08	−0.22 − 0.07	0.31	-0.07	−0.22-0.07	0.33	0.00	−0.14-0.15	0.95

Interaction change x group analyses: Unadjusted; Model 1: adjusted for sex and age (continuous); Model 2: further adjusted for job group (nursery nurse, nursery nurse assistant, other job group), workplace type (integrated, day care, kindergarten), workplace size (continuous), and baseline values of endpoints. Workplace identification number is included in a repeated statement. Statistically significant results are printed in bold

**Table 9:** Intervention effect on workplace social capital in the intervention group (low degree of implementation) compared to the control group during 24 months of follow-up

	n	Unadjusted			Model 1			Model 2		
		Est	95% CI	p	Est	95% CI	p	Est	95% CI	p
Social capital										
Vertical	353	−0.21	−0.56-0.13	0.22	−0.22	− 0.56-0.12	0.20	− 0.21	−0.49-0.08	0.15
Horizontal	353	−0.09	−0.25-0.08	0.29	−0.09	− 0.25-0.08	0.30	− 0.09	−0.24-0.07	0.29

Interaction change x group analyses: Unadjusted; Model 1: adjusted for sex and age (continuous); Model 2: further adjusted for job group (nursery nurse, nursery nurse assistant, other job group), workplace type (integrated, day care, kindergarten), workplace size (continuous), and baseline values of endpoints. Workplace identification number is included in a repeated statement

## Abbreviations

DWECS: The Danish Work Environment Cohort Study
WSC: Workplace social capital
